# Prevalence and Patterns of Chronic Disease Pairs and Multimorbidity among Older Chinese Adults Living in a Rural Area

**DOI:** 10.1371/journal.pone.0138521

**Published:** 2015-09-22

**Authors:** Rui Wang, Zhongrui Yan, Yajun Liang, Edwin C. K. Tan, Chuanzhu Cai, Hui Jiang, Aiqin Song, Chengxuan Qiu

**Affiliations:** 1 Aging Research Center, Department of Neurobiology, Health Care Sciences and Society, Karolinska Institutet-Stockholm University, Stockholm, Sweden; 2 Department of Neurology, Jining No. 1 People’s Hospital, Shandong, China; 3 School of Public Health, Jining Medical University, Shandong, China; 4 Centre for Medicine Use and Safety, Faculty of Pharmacy and Pharmaceutical Sciences, Monash University, Melbourne, Australia; 5 Xing Long Zhuang Hospital, Yankuang Group, Shandong, China; University of Padova, ITALY

## Abstract

**Background:**

The burden of chronic diseases in China is substantial now. Data on patterns of chronic diseases and multimorbidity among older adults, especially among those living in rural areas, are sparse.

**Objective:**

We aim to investigate the prevalence and patterns of chronic disease pairs and multimorbidity in elderly people living in rural China.

**Methods:**

This population-based study included 1480 adults aged 60 years and over (mean age 68.5 years, 59.4% women) living in a rural community. Data were derived from the Confucius Hometown Aging Project in Shandong, China (June 2010-July 2011). Chronic diseases were diagnosed through face-to-face interviews, clinical examinations, and laboratory tests. Patterns of chronic disease pairs and multimorbidity were explored using logistic regression and exploratory factor analyses.

**Results:**

The prevalence of individual chronic diseases ranged from 3.0% for tumor to 76.4% for hypertension, and each disease was often accompanied with three or more other chronic diseases. The observed prevalence of pairs of chronic conditions exceeded the expected prevalence for several conditions, such as cardiovascular diseases and metabolic disorders, as well as pulmonary diseases and degenerative disorders. Chronic multimorbidity (≥2 chronic diseases) affected more than 90% of subjects, and two patterns of chronic multimorbidity were identified: cardiopulmonary-mental-degenerative disorder pattern (overall prevalence, 58.2%), and cerebrovascular-metabolic disorder pattern (62.6%). Prevalence of the cardiopulmonary-mental-degenerative disorder pattern increased with age, and was higher in men than women; whereas prevalence of the cerebrovascular-metabolic disorder pattern was higher in women than in men but did not vary by age.

**Conclusion:**

Chronic multimorbidity was highly prevalent among older Chinese adults living in rural areas, and there were specific patterns of the co-occurrence of chronic diseases. Effort is needed to identify possible preventative strategies based on the potential clustering of chronic diseases.

## Introduction

Chronic non-communicable diseases have replaced infectious diseases to become a major public health burden [1]. Unlike most western countries that have had this transition at a slower pace, China has experienced this shift only in a few decades [2,3], which has consequently caused a rapid increase in chronic disease burden. The proportion of all-cause mortality attributable to chronic disease in China was 58.2% in 1973–1975, and this number increased to 73.8% in 1991 [2]. By 2005, more than 80% of deaths and 70% of disability-adjusted life-years lost were due to chronic diseases in China [2,4]. As many chronic diseases are strongly related to age and often occur concurrently in older people, multimorbidity, defined as the presence of two or more chronic conditions [5], has become increasingly common as people age. Now, China is facing a substantial burden of chronic multimorbidity in the coming decades owing to a rapid increase in the aging population.

The prevalence of multimorbidity has been reported by several population-based studies in China. Specifically, a large-scale population-based study conducted in southern China has recently reported that more than 11% of people of all ages had two or more chronic conditions in 2011 [6]. A systematic review further demonstrated that among older Chinese adults (age ≥60 years), the overall prevalence of multimorbidity was up to 87.0% in urban residents [7]. However, very little information is available regarding the prevalence of multimorbidity in rural China. One population-based study showed that 32% of older adults living in rural areas of Southern China were affected by multimorbidity [8]. Nevertheless, this study included a limited number of chronic diseases (10 chronic health conditions) and all the chronic conditions were ascertained using self-reported questionnaires [8]. Indeed, until recent years, health care service and insurance systems in rural areas were different from those in urban areas. In addition, the prevalence of lifestyle risk factors for chronic diseases such as smoking was higher in rural than urban male residents [9]. Furthermore, research has suggested that in China, certain chronic diseases such as diabetes might have been underdiagnosed among rural residents compared to community-dwelling residents in urban areas [10]. These studies indicate that the burden of chronic diseases in rural areas of China might have been underestimated. More population-based studies carried out in rural China therefore are needed to describe the prevalence and distribution of chronic diseases and multimorbidity using standard assessment approaches.

In addition, diverse patterns of chronic multimorbidity among older adults have been identified in different countries [11–16]. A systematic review revealed three major patterns of multimorbidity in older adults living in Europe, the United States (U.S.), and Australia, which are cardiovascular and metabolic diseases, mental health problems, and musculoskeletal disorders [11]. However, the potential patterns of multimorbidity among older Chinese adults have not yet been reported. Identifying the patterns of chronic multimorbidity is relevant for primary care services. Such knowledge can help policy-makers with proper allocation of resources and delivery of targeted preventative and management services to those most at risk. Thus, in this population-based study, we sought to (1) investigate the prevalence and clustering of chronic diseases (i.e., pairs of chronic diseases and multimorbidity), and (2) identify the patterns of multimorbidity in adults aged 60 years and older living in rural China.

## Materials and Methods

### Study population

This study population included participants from the Confucius Hometown Aging Project (CHAP), as previously described [17,18]. All registered residents who were aged ≥60 years and living in the Xing Long Zhuang community nearby Qufu, Shandong, China, were eligible to participate in this study. The average annual per capita income of rural residents in this area corresponded to the medium level of incomes of rural residents in Shandong province. The CHAP study was conducted by Jining No. 1 People’s Hospital and Jining Medical University in Shandong, China, in collaboration with the Aging Research Center at Karolinska Institutet-Stockholm University in Stockholm, Sweden. CHAP was aimed at investigating cardiovascular risk factors and atherosclerotic mechanisms in aging and health [19].

From June 2010 to July 2011, extensive data were collected through structured interviews, clinical examinations, and laboratory tests. Data collection was carried out by trained nurses, physicians, and laboratory technicians from the Xing Long Zhuang Hospital that provides medical and health care services to residents living in the local community. Of all eligible subjects (n = 1743), 204 refused to participate or moved out of the area, and 59 had missing information on demographics or different health conditions, leaving 1480 subjects for the current analysis.

### Data collection

Epidemiological data were collected using a questionnaire that was developed from the WHO STEPwise approach to surveillance and the Study on Global Ageing and Adult Health [20]. We collected data on age, sex, education, health history (e.g., diabetes, heart disease, and stroke), and use of medications (e.g., antihypertensive agents, blood glucose-lowering drugs, hypolipidemic drugs, antithyroid drugs, and drugs for asthma or tracheitis), as previously reported [19,21]. Weight and height were measured in light clothes without shoes. Body mass index (BMI) was calculated as weight in kilograms divided by height in meters squared. After a 5-min rest, sitting arterial blood pressure (Korotkoff systolic phase I and diastolic phase V) was measured on the right arm using a mercury sphygmomanometer with the cuff maintained at heart level. Blood pressure was measured twice on one occasion, and the mean of the two readings was used in the analysis. The 12-lead resting electrocardiogram was recorded and then analyzed by a physician. Global cognitive function was measured using the Chinese version of the Mini-Mental State Examination (MMSE). Depressive symptoms were assessed using the 15-item Geriatric Depression Scale (GDS-15) [17]. After an overnight fast, a peripheral blood sample was taken at the local hospital. Plasma glucose, total cholesterol, triglycerides, high-density lipoprotein cholesterol (HDL-C), and low-density lipoprotein cholesterol (LDL-C) were measured using an automatic Biochemical Analyzer (Olympus AU400, Olympus Optical Co., Ltd., Tokyo, Japan) at the hospital laboratory.

### Assessment of chronic diseases

Chronic diseases were defined as fulfilling one or more of the following characteristics [22]: permanent conditions, caused by a nonreversible pathological alteration, or required rehabilitation or a long period of care. We ascertained 16 chronic health conditions, as defined below. Hypertension was defined as blood pressure ≥140/90 mmHg or current use of antihypertensive drugs [19,23]; diabetes as fasting plasma glucose ≥7.0 mmol/l or current use of oral blood glucose-lowering medications or insulin injection [24]; obesity as BMI ≥28 kg/m^2^ [19]; and dyslipidemia as total cholesterol >6.2 mmol/l, triglycerides ≥2.3 mmol/l, HDL-C<1.0 mmol/l in men or HDL-C<1.3 mmol/ in women, LDL-C ≥4.1 mmol/l, or use of hypolipidemic drugs [25]. Thyroid dysfunction was ascertained according to self-reported physician diagnosis of hyperthyroidism or hypothyroidism, or use of antithyroid drugs. Coronary heart disease (CHD) and arrhythmia were ascertained according to electrocardiogram or self-reported physician diagnosis of the diseases [26]. Eye problems were ascertained according to abnormal fundus examination or self-reported physician diagnosis of cataract or glaucoma. Chronic obstructive pulmonary disease (COPD) or asthma was ascertained according to self-reported physician diagnosis of the disease, or use of drugs for tracheitis or asthma. Presence of elevated depressive symptoms was defined as the GDS-15 score ≥5 [27]. Cognitive impairment was defined following the education-based cutoffs of MMSE score, i.e., MMSE ≤17 for illiteracy, MMSE ≤20 for primary school (1–6 years), and MMSE ≤24 for middle school and above (≥7 years) [28].

Heart failure, stroke, hearing disorders, tumor, and arthritis were identified according to the self-reported information obtained during the physician interview.

### Statistical analysis

Demographic characteristics of participants by sex were compared using t-test for continuous variables and chi-square test for categorical variables. We present prevalence (per 100 population) of individual chronic diseases and their aggregation (0, 1, 2, or ≥3 chronic diseases), chronic multimorbility (≥2 chronic diseases) and chronic disease pairs. For chronic disease pairs with a frequency >5%, we reported the observed (O) and the expected (E) prevalence of the disease pairs and their ratio (O/E), in which the expected prevalence of the disease pairs was calculated as (prevalence of disease A)×(prevalence of disease B) [22,29]. Logistic regression models were used to analyze the association between two chronic diseases in the pairs in the univariate model as well as in the model that was controlled for demographics and other chronic diseases. To identify patterns of chronic multimorbidity, exploratory factor analysis was performed using a tetrachoric correlation matrix. The pattern (or a cluster) of chronic health conditions was determined if the eigenvalue was greater than 1 and if the factor loading for a chronic disease was ≥0.25. When a certain disease has the factor loading ≥0.25 in more than one factor, this disease was clustered into the group with a larger factor loading value. We reported the oblique (oblimin) rotated factor loadings for individual chronic diseases of each pattern, which indicates the strength and direction of a factor contributing to the pattern of multimorbidity [29]. We reported the age- and sex-specific prevalence of multimorbidity overall and by different patterns.

Stata version 12.0 for Windows (StataCorp 2011, College Station, TX: StataCorp LP) was used for all analyses.

### Ethics approval

The CHAP protocols were reviewed and approved by the Ethics Committee at Jining No. 1 People’s Hospital of Jining Medical University, Shandong, China. We obtained written informed consent from all participants. In the case of cognitively impaired persons, written informed consent was obtained from relatives of participants. Research within CHAP was conducted following the Ethical Principles for Medical Research Involving Human Subjects expressed in the Declaration of Helsinki.

## Results

### Demographic characteristics

Table 1 shows the demographic characteristics of the study participants by sex. The mean age of the 1480 participants was 68.5 (standard deviation [SD] 5.0) years, 59.4% were women, and 31.1% did not attend any formal school. Men had a higher level of education than women (*P*<0.01).

**Table 1:** Demographic characteristics of the study participants.

Characteristics	Total (n = 1480)	Men (n = 601)	Women (n = 879)	P-value^a^
Age (years), mean (SD)	68.5 (5.0)	68.7 (4.9)	68.5 (5.0)	0.47
Age groups, n (%)				
60–64	364 (24.6)	145 (24.1)	219 (24.9)	
65–69	471 (31.8)	176 (29.3)	295 (33.6)	
70–74	469 (31.7)	204 (33.9)	265 (30.2)	
≥75	176 (11.9)	76 (12.7)	100 (11.4)	0.24
Education level, n (%)				
No formal school	460 (31.1)	76 (12.6)	384 (13.7)	
Primary school	706 (47.7)	312 (51.9)	394 (44.8)	
Middle school	205 (13.9)	138 (23.0)	67 (7.6)	
High school or above	109 (7.4)	75 (12.5)	34 (3.9)	<0.01

^a^P-value was for test of sex difference.

Abbreviation: SD, Standard Deviation.

### Prevalence of individual chronic diseases and their clusters

Of the 16 chronic diseases that were examined in this study, the overall prevalence of chronic diseases ranged from 3.0% for tumor to 76.4% for hypertension. Of the 1480 participants, only 1.5% did not have any of the examined chronic diseases, 8.0% had only one chronic disease, 13.8% had two chronic diseases, and 76.7% had three or more chronic diseases. The average number of chronic diseases that were present in each individual was 4.1 (SD 2.1). There was no significant sex difference in the overall clustering of chronic diseases.

**Fig 1:**
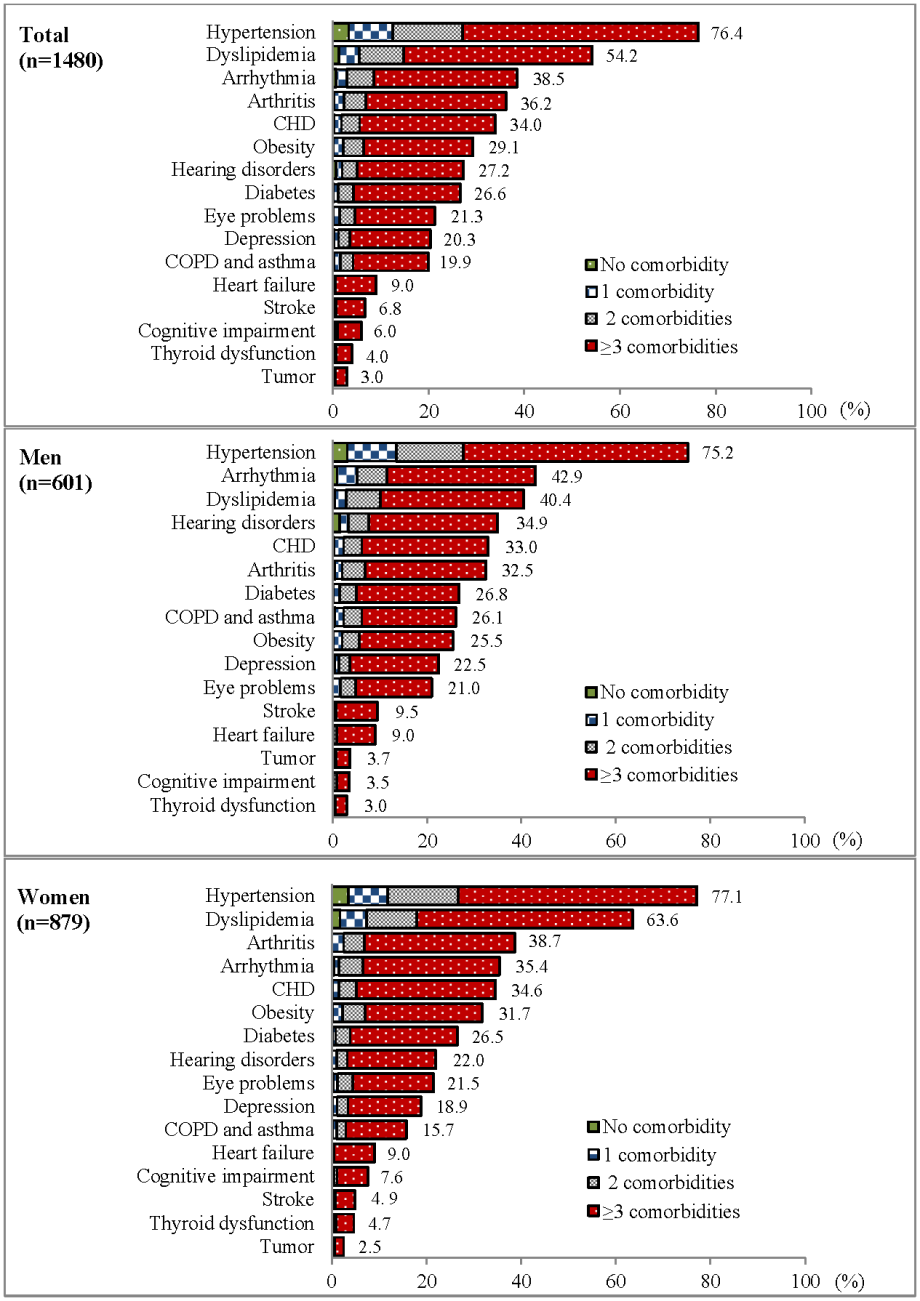
Prevalence (per 100) and co-occurrence of chronic diseases (n = 1480). Abbreviations: COPD, chronic obstructive pulmonary disease; CHD, coronary heart disease.

Fig 1 shows the prevalence of individual chronic diseases with different numbers of comorbid diseases. Overall, the top five most common chronic diseases were hypertension (76.4%), dyslipidemia (54.2%), arrhythmia (38.5%), arthritis (36.2%), and CHD (34%). More than 96% of persons with the most prevalent chronic diseases (e.g., hypertension, dyslipidemia, and arrhythmia) had at least one comorbid disease.

### Prevalence and association of chronic disease pairs

Table 2 shows the observed and expected prevalence of pairs of chronic conditions, and their associations (odds ratio [OR] and 95% confidence interval [CI]). The most prevalent chronic disease pair was hypertension and dyslipidemia (46.9%), followed by hypertension and CHD (28.0%), hypertension and obesity (24.7%), and hypertension and diabetes (22.4%). The least prevalent chronic disease pair was heart failure and arthritis (5.1%). The most common form of concurrently having three chronic disorders was hypertension, dyslipidemia, and arrhythmia (overall 18.3%, 16.1% in men, and 19.8% in women).

**Table 2:** Observed and expected prevalence of frequent chronic disease pairs and their associations.

Pairs of co-occurring chronic diseases by disease groups	No. of disease pairs	Prevalence, %			Odds ratio (95% CI)	
		O	E	O/E	Crude	Adjusted^a^
**Hypertension group**						
Hypertension and dyslipidemia	694	46.9	41.4	1.1	1.7 (1.4–2.2)**	1.5 (1.1–1.9)**
Hypertension and arthritis	419	28.3	27.6	1.0	1.2 (0.9–1.5)	1.1 (0.9–1.5)
Hypertension and CHD	415	28.0	25.9	1.1	1.8 (1.3–2.3)**	1.5 (1.1–2.0)**
Hypertension and hearing disorder	306	20.7	20.8	1.0	1.0 (0.7–1.3)	0.9 (0.6–1.2)
Hypertension and eye problem	244	16.5	16.2	1.0	1.1 (0.8–1.5)	1 (0.7–1.3)
Hypertension and stroke^b^	91	6.1	5.2	1.2	3.3 (1.7–6.7)**	**2.7 (1.3–5.6)** **
Hypertension and heart failure	111	7.5	6.9	1.1	1.6 (1.0–2.6)*	1.3 (0.8–2.1)
Hypertension and obesity	365	24.7	22.3	1.1	2.0 (1.5–2.7)**	1.9 (1.4–2.6)**
Hypertension and COPD/asthma	214	14.5	15.2	1.0	0.8 (0.6–1.0)^+^	0.7 (0.5–0.9)*
Hypertension and depression	242	16.4	15.5	1.1	1.3 (1.0–1.8)^+^	1.2 (0.8–1.6)
Hypertension and diabetes	331	22.4	20.3	1.1	1.9 (1.4–2.6)**	1.5 (1.1–2.1)**
Hypertension and arrhythmia	450	30.4	29.4	1.0	1.3 (1.0–1.7)^+^	1.1 (0.9–1.5)
**Dyslipidemia group**						
Dyslipidemia and arthritis	311	21.0	19.6	1.1	1.3 (1.0–1.6)*	1.1 (0.9–1.4)
Dyslipidemia and arrhythmia	322	21.8	20.8	1.0	1.2 (0,9–1,4)	1.2 (0.9–1.5)
Dyslipidemia and heart failure	84	5.7	4.9	1.2	1.5 (1.0–2.2)*	1.1 (0.8–1.7)
Dyslipidemia and obesity	274	18.5	15.8	1.2	1.7 (1.4–2.2)**	1.5 (1.1–1.9)**
Dyslipidemia and COPD/asthma	160	10.8	10.8	1.0	1.0 (0.8–1.3)	1.1 (0.9–1.5)
Dyslipidemia and CHD	305	20.6	18.4	1.1	1.5 (1.2–1.9)**	1.2 (1.0–1.5)
Dyslipidemia and hearing disorder	214	14.5	14.8	1.0	0.9 (0.7–1.2)	1.0 (0.8–1.3)
Dyslipidemia and diabetes^b^	266	18.0	14.4	1.2	2.1 (1.7–2.7)**	**2.0 (1.5–2.6)** **
Dyslipidemia and depression	187	12.6	11.0	1.1	1.5 (1.2–1.9)**	1.5 (1.1–1.9)**
Dyslipidemia and eye problem	173	11.7	11.5	1.0	1.0 (0.8–1.3)	0.9 (0.7–1.2)
**Obesity group**						
Obesity and COPD/asthma	81	5.5	5.8	0.9	0.9 (0.7–1.2)	1 (0.7–1.4)
Obesity and depression	91	6.1	5.9	1.0	1.1 (0.8–1.4)	1 (0.8–1.4)
Obesity and diabetes	146	9.9	7.8	1.3	1.6 (1.3–2.1)**	1.5 (1.2–2.0)**
Obesity and hearing disorder	118	8.0	7.9	1.0	1.0 (0.8–2.1)	1.1 (0.8–1.4)
Obesity and CHD	168	11.4	9.9	1.1	1.4 (1.1–1.7)*	1.3 (1.0–1.7)*
Obesity and arthritis	173	11.7	10.6	1.1	1.3 (1.0–1.6)*	1.2 (0.9–1.6)
Obesity and arrhythmia	163	11.0	11.2	1.0	1.0 (0.8–1.2)	1.0 (0.7–1.2)
**Diabetes group**						
Diabetes and depression	88	5.9	5.4	1.1	1.2 (0.9–1.6)	1.0 (0.8–1.4)
Diabetes and eye problem	107	7.2	5.7	1.3	1.6 (1.2–2.1)**	1.6 (1.2–2.2)**
Diabetes and hearing disorder	111	7.5	7.2	1.0	1.1 (0.8–1.4)	1.0 (0.8–1.4)
Diabetes and CHD	165	11.1	9.0	1.2	1.6 (1.3–2.0)**	1.4 (1.1–1.8)*
Diabetes and arthritis	144	9.7	9.6	1.0	1.0 (0.8–1.3)	0.9 (0.7–1.1)
Diabetes and arrhythmia	160	10.8	10.2	1.1	1.1 (0.9–1.4)	1.0 (0.7–1.2)
**Heart diseases group**						
Heart failure and CHD^b^	89	6.0	3.0	2.0	4.6 (3.1–6.7)**	**3.1 (2.1–4.7)** **
Heart failure and arthritis	75	5.1	3.2	1.6	2.5 (1.7–3.6)**	1.7 (1.1–2.5)*
Heart failure and arrhythmia^b^	98	6.6	3.5	1.9	5.2 (3.5–7.8)**	**4.2 (2.7–6.3)** **
CHD and arthritis	230	15.5	12.3	1.3	1.9 (1.5–2.3)**	1.6 (1.3–2.0)**
CHD and arrhythmia	253	17.1	13.0	1.3	2.1 (1.7–2.7)**	1.8 (1.4–2.2)**
CHD and hearing disorder	155	10.5	9.2	1.1	1.3 (1.0–1.7)*	1.0 (0.7–1.3)
CHD and eye problem	122	8.2	7.2	1.1	1.3 (1.0–1.7)*	1.0 (0.8–1.3)
CHD and depression	125	8.4	6.9	1.2	1.5 (1.2–2.0)**	1.2 (0.9–1.6)
CHD and COPD/asthma	122	8.2	6.8	1.2	1.5 (1.1–1.9)**	1.2 (0.9–1.7)
Arrhythmia and hearing disorder	179	12.1	10.5	1.2	1.3 (1.0–1.7)^+^	1.1 (0.9–1.5)
Arrhythmia and arthritis	228	15.4	13.9	1.1	1.3 (1.0–1.7)*	1.1 (0.8–1.4)
Arrhythmia and eye problem	141	9.5	8.2	1.2	1.4 (1.1–1.8)*	1.2 (0.9–1.6)
Arrhythmia and depression	133	9.0	7.8	1.1	1.3 (1.1–1.6)*	1.1 (0.8–1.4)
Arrhythmia and COPD/asthma	128	8.6	7.7	1.1	1.4 (1.1–1.8)**	1.0 (0.7–1.3)
**Hearing, eyes, depression, and COPD/asthma group**						
COPD/asthma and depression	86	5.8	4.1	1.4	1.9 (1.4–2.5)**	1.4 (1.1–2.0)*
COPD/asthma and eye problem	82	5.5	4.2	1.3	1.6 (1.2–2.1)**	1.3 (1.0–1.8)^+^
COPD/asthma and hearing disorder	128	8.6	5.4	1.6	2.5 (1.9–3.3)**	1.8 (1.3–2.4)**
COPD/asthma and arthritis	144	9.7	7.2	1.4	1.9 (1.5–2.5)**	1.7 (1.3–2.2)**
Depression and hearing disorder	114	7.7	5.5	1.4	1.9 (1.4–2.5)**	1.5 (1.1–2.0)*
Depression and arthritis	132	8.9	7.4	1.2	1.5 (1.2–1.9)**	1.2 (0.9–1.6)
Eye problem and hearing disorder	106	7.2	5.8	1.2	1.5 (1.1–1.9)**	1.2 (0.9–1.6)
Eye problem and arthritis	142	9.6	7.7	1.2	1.6 (1.3–2.1)**	1.5 (1.1–1.9)**
Hearing disorder and arthritis^b^	213	14.4	9.8	1.5	2.6 (2.1–3.1)**	**2.7 (2.1–3.4)** **

Abbreviations: CHD, coronary heart disease; COPD, chronic obstructive pulmonary disease; O, observed prevalence; E, expected prevalence.

^a^Odds ratio was controlled for demographics and other chronic diseases.

^b^The five pairs of chronic diseases that showed the strongest association after adjusting for demographics and other chronic diseases.

*P<0.05,

**P<0.01.

Heart failure and arrhythmia, heart failure and CHD, hearing disorder and arthritis, hypertension and stroke, and dyslipidemia and diabetes, were the five pairs of chronic diseases with the strongest associations; the adjusted OR (95% CI) of their associations ranged from 2.0 (1.5–2.6) for the dyslipidemia and diabetes pair to 4.2 (2.7–6.3) for the heart failure and arrhythmia pair. Hypertension and pulmonary disorder (COPD and asthma) constituted the only pair with a significantly inverse correlation (adjusted OR 0.7; 95% CI 0.5–0.9).

### Patterns of chronic multimorbidity

We identified two patterns of chronic multimorbidity, which explained 43.7% of the total variance (Table 3). The pattern 1 (eigenvalue 2.42) was characterized by cardiopulmonary disorders (heart failure, arrhythmia, coronary heart diseases, COPD and asthma), depression, and degenerative disorders (eye problems and hearing disorders), whereas the pattern 2 (eigenvalue 1.34) was characterized by stroke and metabolic factors (hypertension, diabetes, dyslipidemia, and obesity).

**Table 3:** Rotated loadings for each of the chronic diseases in factors with an eigenvalue greater than 1 from factor analysis (n = 1480).

Parameters and diseases	Factor 1	Factor 2
Eigenvalue	2.42	1.34
Cumulative percent	28.2	43.7
Hearing disorders	**0.45**	-0.08
Depression	**0.36**	0.13
Eye problems	**0.31**	-0.02
COPD and asthma	**0.46**	-0.18
Arthritis	**0.46**	-0.02
Arrhythmia	**0.43**	0.09
Heart failure	**0.76**	0.12
Coronary heart disease	**0.46**	0.27
Stroke	0.20	**0.40**
Hypertension	0.08	**0.54**
Diabetes	0.10	**0.47**
Dyslipidemia	0.12	**0.43**
Obesity	-0.06	**0.35**
Thyroid dysfunction	0.23	-0.06
Tumour	0.07	0.01
Cognitive impairment	0.17	0.14

Notes. Chronic diseases with a factor loading <0.25 were omitted for further clustering of multimorbidity patterns. Abbreviation: COPD, chronic obstructive pulmonary disease.

### Prevalence of chronic multimorbidity

**Fig 2:**
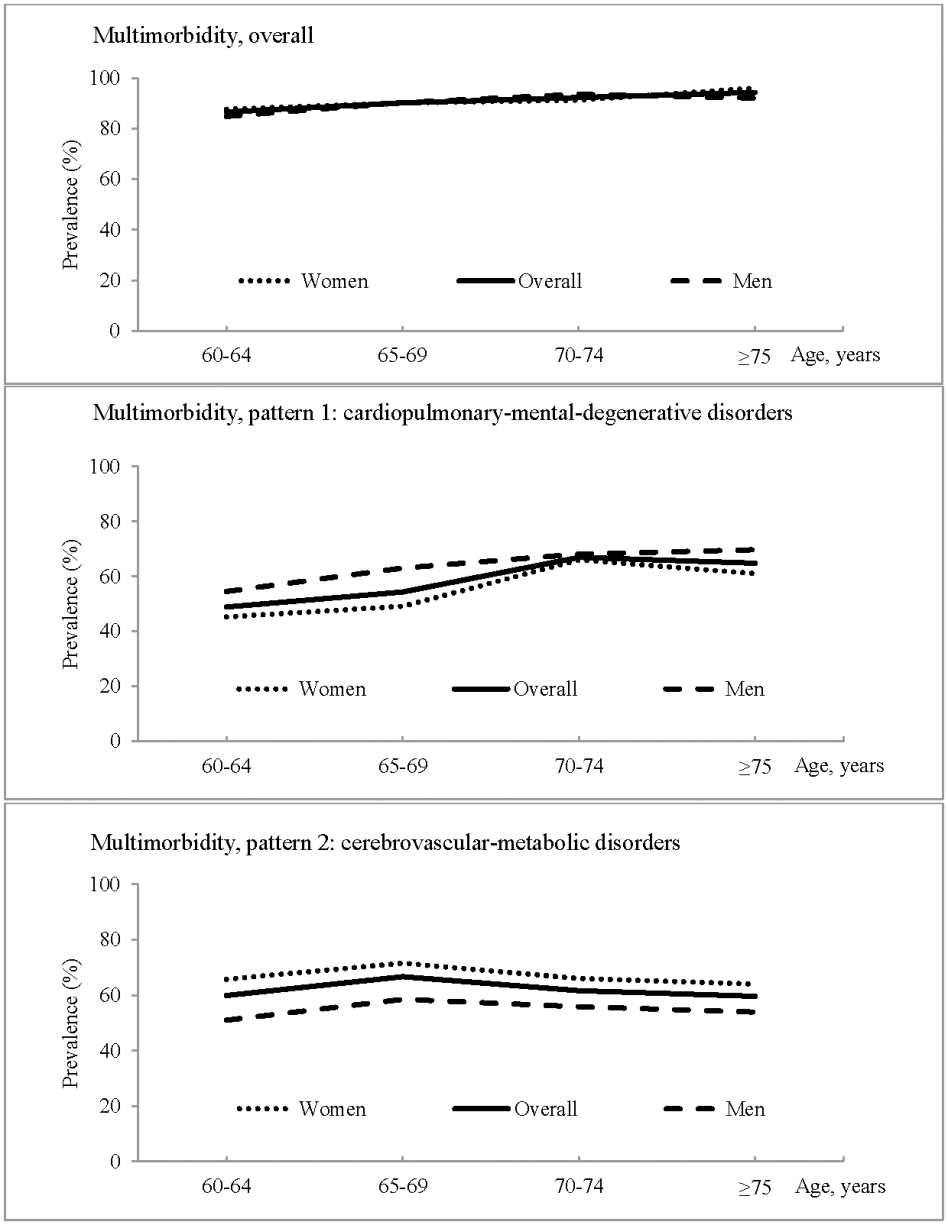
Age- and sex-specific prevalence of chronic multimorbidity and two patterns of multimorbidity (n = 1480).

The overall prevalence of chronic multimorbidity was 90.5% (men 90.4%, women 90.6%, *P* = 0.89); the prevalence was 58.2% (men 63.6%, women 54.6%, *P*<0.01) for the cardiopulmonary-mental-degenerative disorder pattern, and 62.6% (men 55.2%, women 67.6%, *P*<0.01) for the cerebrovascular-metabolic disorder pattern. Fig 2 shows the age- and sex-specific prevalence of chronic multimorbidity overall and by two patterns. Firstly, the prevalence of overall multimorbidity increased slightly with advancing age, with no apparent difference by sex. Secondly, the prevalence of the cardiopulmonary-mental-degenerative disorder pattern also increased with age, and this pattern appeared to be more prevalent in men than in women. Thirdly, the prevalence of the cerebrovascular-metabolic disorder pattern was largely constant across the age groups, whereas women showed higher prevalence than men in all age groups.

The demographic-adjusted OR (95% CI) for overall multimorbidity related to every 5-year increase in age was 1.07 (1.03–1.11); the corresponding figures were 1.06 (1.04–1.09) for the multimorbidity pattern of cardiopulmonary-mental-degenerative disorders and 0.99 (0.97–1.02) for the cerebrovascular-metabolic disorder pattern. There was no significant sex difference in the prevalence of overall multimorbidity (men vs. women: adjusted OR 0.98; 95% CI 0.67–1.44), whereas men were more likely than women to have the multimorbidity pattern characterized by cardiopulmonary-mental-degenerative disorders (men vs. women: adjusted OR 1.50; 95% CI 1.18–1.92) and women were more likely than men to have the multimorbidity pattern of cerebrovascular-metabolic disorders (women vs. men: adjusted OR 1.68; 95% CI 1.32–2.15).

## Discussion

We explored the prevalence and patterns of chronic disease pairs and multimorbidity among older Chinese adults living in a rural community. The main findings from this study are summarized as follows: (1) Chronic multimorbidity was highly prevalent that affected more than 90% of older adults; the average number of chronic diseases concurrently occurring in a person was around four; (2) certain chronic diseases often occurred in pairs, especially those pairs involving hypertension or dyslipidemia; (3) we identified two patterns of chronic multimorbidity: the cardiopulmonary-mental-degenerative disorder pattern, which increased with age and was more prevalent in men than in women; and the cerebrovascular-metabolic disorder pattern, which was largely constant with age and more prevalent in women than in men.

The prevalence of chronic multimorbidity among elderly people varies widely across countries and areas, depending on age of study population, number of involved chronic diseases, measurements, and sampling frame [6]. In European countries, the prevalence of multimorbidity among elderly people ranged from 13.1% to 98.0%, and the variability regarding the numbers and types of the chronic diseases was very wide [13,14]. A systematic review showed a list of 20 chronic diseases that were frequently involved in the previous studies in western societies, including cardiovascular diseases (stroke, coronary heart diseases, heart failure, and cardiac arrhythmia), cardiometabolic and endocrine disorders (hypertension, diabetes, obesity, dyslipidemia, and thyroid disease), pulmonary disorders (COPD and asthma), mental and cognitive diseases (depression, dementia, and anxiety), degenerative disorders (joint disease and hearing problems), cancer, anemia, prostatic hypertrophy, and osteoporosis [11]. Involving the majority of these chronic diseases, we observed that the prevalence of multimorbidity in a rural community in China was over 90%, and more than three quarters of older adults had three or more chronic diseases. These figures were relatively low in other studies of older adults living in urban China. For instance, the overall prevalence of multimorbidity varied from 6.4% to 87.0%; and the prevalence of having at least three chronic diseases ranged from 2.5% to 54.9% [7]. In one study involving older adults living in a rural area of Southern China, the prevalence of chronic multimorbidity was reported at 32%, and 13.5% had three or more chronic diseases [8]. Compared with other studies conducted in China, the higher prevalence of chronic multimorbidity in our study sample may be due to our employment of a broader range of chronic health conditions, as well as the more comprehensive diagnostic approaches. For example, previous studies have tended to rely on self-reported data, which may be problematic in older adults living in rural areas with a lower level of education and being less aware of chronic medical conditions, such as hypertension, dyslipidemia, and diabetes. Using comprehensive diagnostic approaches would improve the detection rate of these diseases. Additionally, the differences in the prevalence of chronic multimorbidity between urban and rural areas may be attributed to an imbalance in health care resource allocation and health insurance systems.

When we evaluated the associations between the two diseases of common co-occurring chronic conditions, we detected that five pairs of chronic diseases (heart failure and arrhythmia, heart failure and CHD, hearing disorders and arthritis, hypertension and stroke, and dyslipidemia and diabetes) were each highly correlated. This was consistent with a Swedish study which demonstrated a strong link among different heart diseases (i.e., heart failure and CHD, and heart failure and atrial fibrillation) [22], and a German study also observed that hypertension and stroke, as a common pair of chronic diseases, were greatly associated, as expected [29]. Interestingly, previous studies showed that the association between hypertension and stroke was even stronger in Chinese populations than in European populations [30,31]. In agreement with our finding regarding the co-occurrence of dyslipidemia and diabetes, a U.S. national study of elderly nursing home residents revealed that older adults with diabetes were significantly more likely to have certain chronic conditions simultaneously, including dyslipidemia [32]. The observed co-occurrence of hearing disorders and arthritis was much higher than their expected co-occurrence. One possibility is that rheumatoid arthritis may involve multifocal locations in audiologic systems, which can cause a mixed type of hearing loss [33]. Finally, evidence has shown that cardiovascular diseases are the leading cause of death among those with pulmonary disorders [34,35]. Thus potential survival bias may have explained why we did not observe any significant association of chronic disease pairs between pulmonary disorders and cardiovascular diseases, and even an inverse correlation between hypertension and pulmonary disorders.

Two patterns of chronic multimorbidity were observed in our study. As the majority of cases of arthritis are osteoarthritis, which is a common degenerative joint disease among older adults [36], arthritis, together with eye problems and hearing disorders, were included in the degenerative disorder group. In contrast to findings from previous studies conducted in Europe and the U.S. [22,29,37,38], we detected a pattern that covers cardiovascular diseases, pulmonary disorders, mental disease, and degenerative disorders. We revealed that the prevalence of multimorbidity in this pattern increased with age, and was higher among men than women. These findings are in line with previous epidemiologic data of chronic diseases in Chinese populations. For instance, earlier studies showed that the prevalence of cardiopulmonary diseases (coronary heart diseases and COPD) increased with age, and was higher in men than in women [39,40]; the prevalence of depression was higher in rural than in urban settings, and increased slightly with advancing age [41,42]; and degenerative disorders (e.g., eye and hearing disorders) were associated with an increased likelihood of depression among older Chinese adults [43]. Metabolic disorders and stroke constituted the second pattern of chronic multimorbidity. In contrast to the first pattern, the prevalence of multimorbidity in cerebrovascular-metabolic disorders was higher among women than men. This sex difference in the distribution of metabolic risk factors (e.g., obesity and diabetes) among older adults was also highlighted in previous Chinese studies [44,45]. A reduction in post-menopausal estrogen production may partly explain this sex difference, as it can increase total cholesterol levels and insulin-resistance [46]. Moreover, extending the results of the pair-wise association between hypertension and stroke mentioned above, the identification of the multimorbidity pattern of cerebrovascular-metabolic disorders provides further evidence to support the clustering of metabolic disorders and stroke, suggesting the need for multi-targeted prevention and management strategies for stroke in China, especially in rural areas. Metabolic risk factors are known to be associated with both cerebrovascular and heart disorders. Although metabolic risk factors were clustered with cerebrovascular disease, it is notable that the factor loading for coronary heart disease contributing to the chronic multimorbidity pattern of cerebrovascular-metabolic disorders (factor 2) was 0.27, which is higher than the initial criteria (i.e., >0.25) for being clustered with the chronic multimorbidity pattern that included metabolic factors, suggesting that metabolic risk factors were also closely associated with coronary heart disease.

This population-based study targeted elderly people living in a rural region in China to whom very little attention has been paid to by the research community. Moreover, our study covered a broad range of chronic health conditions in which most of these conditions were determined by integrating information from interviews, clinical examinations, instrumental and laboratory tests. However, our study has limitations. Firstly, five chronic diseases (heart failure, stroke, hearing disorders, tumor, and arthritis) were ascertained only based on self-report, which may be less accurate in older adults with cognitive impairment. Secondly, our study sample was relatively young (mean age 68.6 years), and the oldest-old (age ≥80 years) accounted for only a small proportion (2.2%) of the total sample. Thus, caution should be taken when comparing our results with those from very old cohorts. Finally, the cross-sectional association for chronic disease pairs may be subject to bias owing to selective survival, especially when some diseases (e.g., stroke and heart failure) are highly related to attrition.

## Conclusion

In conclusion, chronic multimorbidity was highly prevalent among older Chinese adults living in rural areas. Additionally, two prevalent patterns of chronic multimorbidity, the cardiopulmonary-mental-degenerative disorder pattern and the cerebrovascular-metabolic disorder pattern, with different distributions by age and sex were observed among older adults living in rural China. Findings from this study have significant implications for primary care planning, general practitioners, and policy-makers, especially with regards to chronic disease management and the delivery of primary care services to older people residing in rural China. Future research should further explore the potential risk factors or determinants for these two patterns of chronic multimorbidity in older adults.

## Supporting Information

S1 Table Detailed figures of prevalence and co-occurrence of chronic diseases in the CHAP sample (per 100 population). (XLSX)
